# Impact of Concurrent Coincident Use of Metformin During Lung Stereotactic Body Radiation Therapy

**DOI:** 10.7759/cureus.14157

**Published:** 2021-03-28

**Authors:** Kyle Stang, Fiori Alite, William Adams, Basel Altoos, Christina Small, Edward Melian, Bahman Emami, Matthew Harkenrider

**Affiliations:** 1 Radiation Oncology, Loyola University Chicago Stritch School of Medicine, Maywood, USA; 2 Radiation Oncology, Geisinger Cancer Institute, Danville, USA; 3 Public Health Sciences, Loyola University Chicago, Chicago, USA; 4 Radiation Oncology, Loyola University Medical Center, Maywood, USA

**Keywords:** metformin, lung cancer, sbrt

## Abstract

Introduction

Recent data suggest synergy of chemoradiotherapy and metformin in locally-advanced non-small cell lung cancer (NSCLC). It remains unclear if similar synergy exists with stereotactic lung body radiation therapy (SBRT) and metformin. We analyzed the role of metformin on progression-free survival (PFS) and toxicity in the setting of lung SBRT.

Methods

We identified 31 patients on metformin-treated with SBRT for early-stage NSCLC. Eighty-nine similarly treated patients were chosen as controls. Kaplan-Meier method was used to estimate cumulative PFS probabilities.

Results

Median follow-up was 30.7 months. Forty-two patients had diabetes, 31 (74%) of which were taking metformin concurrent with SBRT. Median PFS for metformin-users vs. metformin non-users was 36.4 months vs 48.9 months, respectively (p = 0.29). Among diabetic patients, median PFS for metformin users was 36.4 months and was unobserved for non-users (p= 0.40). On univariable analysis, male sex (p = 0.03) and tumor size (p = 0.01) were associated with the risk of progression or death; use of metformin was not significant (p = 0.34). There was no difference in grade ≥2 radiation pneumonitis between metformin users vs non-users (p = 0.51)

Conclusion

In this retrospective sample of lung SBRT patients, we did not detect a meaningful effect of concurrent metformin use on PFS. Since SBRT and conventional RT may have different cell kill mechanisms, the previously described beneficial effects of metformin may not apply in a hypofractionated setting. These results should be validated in an independent dataset, and we await the results of ongoing clinical trials.

## Introduction

There will be an estimated 234,030 newly diagnosed cases and 154,050 deaths from lung cancer in the United States in 2018, representing 14% of all new cancer diagnoses and 26% of cancer deaths [1]. Significant advances have been made in chemotherapy and radiation therapy delivery, which have contributed to decreasing the mortality rate of lung cancer; however, outcomes remain sub-optimal, with a 5-year survival of Stage I-II non-small cell lung cancer (NSCLC) of 52% [2]. 

Despite substantial research and development, poor outcomes have driven investigation into potential adjunctive agents, such as mutation-driven targeted biologic therapy and immunotherapy [3]. Interestingly, recent data have demonstrated the antineoplastic effects of oral anti-hyperglycemic agents [4]. In particular, metformin is one of the most widely prescribed first-line oral diabetic medications in the world [5]. In preclinical data, metformin has been shown to have cytotoxic and tumour suppressor effects in several cancer cell lines [6]. Additionally, studies have established metformin as having radiosensitization properties [7]. In retrospective population studies, metformin use has been associated with lower cancer incidence and improved cancer outcomes in several disease sites [8].

In NSCLC cell lines specifically, multiple reports have shown that metformin directly exhibits antineoplastic properties and acts as a radiosensitizer [9,10]. Two retrospective studies investigated the effect of concurrent metformin use with chemoradiotherapy for locally advanced NSCLC. One study found an improved distant metastasis-free survival (DMFS) and progression-free survival (PFS) in the metformin-user cohort, but no difference in overall survival (OS) [11]. The second study found no difference in survival or failure patterns between metformin users and non-users [12].

Conversely, other retrospective studies have found a decreased risk of lung cancer in diabetic patients taking metformin vs those not on metformin, as well as improved overall survival in patients who received first-line chemotherapy for lung cancer concurrently with metformin vs insulin or other antidiabetic medications [13,14].

Prospective, randomized trials have accrued or are currently accruing for locally advanced NSCLC, (NCT02186847, NCT02115464), randomizing to chemoradiotherapy without metformin, notably NRG LU001, and data are currently maturing [15,16]. It remains unclear what role metformin has in influencing cancer outcomes in the setting of SBRT for early-stage NSCLC. This study aims to investigate the effect of concurrent metformin use on PFS and toxicity in a retrospective sample of patients treated with SBRT for early-stage NSCLC.

## Materials and methods

Patient Population

The study received Institutional Review Board (IRB) approval before beginning any study procedures. From our institutional lung SBRT database, we identified 31 patients who were taking metformin during the time of treatment. Eighty-nine additional non-metformin users with adequate follow-up were chosen as the control group. Metformin use was defined as being actively prescribed within one week of SBRT treatment. Medication profiles for patients were retrieved from the electronic medical records. Diabetic patients were defined as those patients who diagnosed with diabetes mellitus in electronic medical records before radiation oncology consultation.

Patient Treatment and Follow-up

Patients were planned with 4-dimensional computed tomography (CT) simulation. Tumours were contoured on the free-breathing and maximum intensity projection (MIP) image datasets and identified as the internal target volume (ITV). A 5mm uniform planning target volume (PTV) margin was placed around the ITV. Linear accelerator-based SBRT was delivered to a dose of 50-60 Gy in 5 fractions for all patients. Plans were prospectively evaluated for normal tissue sparing and, after 2008, plans were evaluated as per Radiation Therapy Oncology Group (RTOG) 0813 criteria for normal tissue sparing [17]. Overall treatment was completed within 1-3 weeks from the start date. Patients at our institution treated with SBRT for early-stage NSCLC were treated in a consecutive-day fractionation schedule before 2009 and non-consecutive schedule after that. Follow-up protocol consisted of chest CT and evaluation of toxicity at 6-12 weeks then every 3-6 months for one year, every six months until five years, then annually. Toxicity was scored according to Common Terminology Criteria for Adverse Events version 4.0. Local control was classified by the Radiation Therapy Oncology Group RTOG 0236 criteria and defined as the absence of local failure. Local failure was defined as at least 20% increase in the largest dimension of treated tumour measurable by CT and positron emission tomography (PET) imaging with standard uptake value of similar or greater intensity as the pretreatment staging PET, or the measurable tumour with biopsy-confirmed viable carcinoma [18].

Statistical Analysis

Pearson chi-square tests were used to assess associations between metformin and disease progression, diabetes status, T-stage, sex, treatment era, pathology, mortality, and dose. In these comparisons, expected frequencies were monitored, and Fisher’s exact tests were used when such values were sparse. Independent samples t-tests were used to compare the distributions of tumour size and age between those using and not using metformin while a non-parametric Wilcoxon rank-sum test was used to compare the Karnofsky Performance Score (KPS) between the two cohorts.

Univariable Cox proportional hazards models were used to estimate the risk of disease progression or mortality as a function of patients’ metformin use, diabetes status, tumour size, T-stage, sex, age, treatment era, KPS score, pathology, and dose. In these models, the proportional hazards assumption was assessed graphically using Martingale residuals as described by Lin, Wei, and Ying [19]. Finally, a reverse Kaplan-Meier method was used to estimate median follow-up time (as described by Schemper and Smith). Traditional Kaplan-Meier methods were used to estimate the median progression-free survival time in months [20]. Generalized Wilcoxon tests were used to compare survival distributions among patients with diabetes who use and do not use metformin. All analyses and figures were completed using SAS version 9.4 (Cary, NC).

## Results

One hundred and twenty patients treated with SBRT for early-stage NSCLC were included in the analysis. Forty-two patients had diabetes, of which 31 (74%) were taking metformin concurrent with SBRT. A summary of patient characteristics is shown in Table 1. The median follow-up was 30.7 (95% CI: 26.87 - 34.14) months.

**Table 1:** Patient characteristics by metformin status

	Total (N = 120)	Use of Metformin		p
		Yes (n = 31)	No (n = 89)	
Disease Progression	16 (13%)	7 (23%)	9 (10%)	.12
Diabetes	42 (35%)	31 (100%)	11 (12%)	.001
T-Stage				.02
1a or 1a	95 (79%)	29 (94%)	66 (74%)	
2a or 2b	25 (21%)	2 (6.5%)	23 (26%)	
Male Sex	80 (67%)	17 (55%)	63 (71%)	.10
Treatment Era				.98
Before 2009	23 (19%)	6 (19%)	17 (19%)	
After 2009	97 (81%)	25 (81%)	72 (81%)	
Pathology				.70
Adenocarcinoma	40 (33%)	12 (39%)	28 (31%)	
Squamous	31 (26%)	9 (29%)	22 (25%)	
NSCLC NOS	17 (14%)	4 (13%)	13 (15%)	
No pathology	32 (27%)	6 (19%)	26 (29%)	
Deceased	49 (41%)	13 (42%)	36 (40%)	.88
Mean Tumor Size (SD)	2.24 (1.04)	2.09 (0.85)	2.29 (1.10)	.35
Mean Age (SD)	71.38 (9.09)	71.90 (8.01)	71.20 (9.48)	.71
Median KPS (IQR)	80 (70 – 90)	80 (70 – 90)	80 (70 – 90)	.83
Dose				.78
50 Gy x 5	102 (85%)	26 (84%)	76 (85%)	
60 Gy x 5	18 (15%)	5 (16%)	13 (15%)	
Note: KPS = Karnofsky Performance Score. NSCLC NOS = Non-small cell lung cancer not otherwise specified. SD = Standard deviation of the mean. IQR = Interquartile range.				

The most common metformin dose was 500mg BID, with a range total cumulative daily dose of 500mg to 2,000mg. Metformin users were significantly less likely to have T-stage 2a or 2b than non-Metformin users (p =0.02); otherwise, patients were well balanced on all other characteristics.

**Figure 1:**
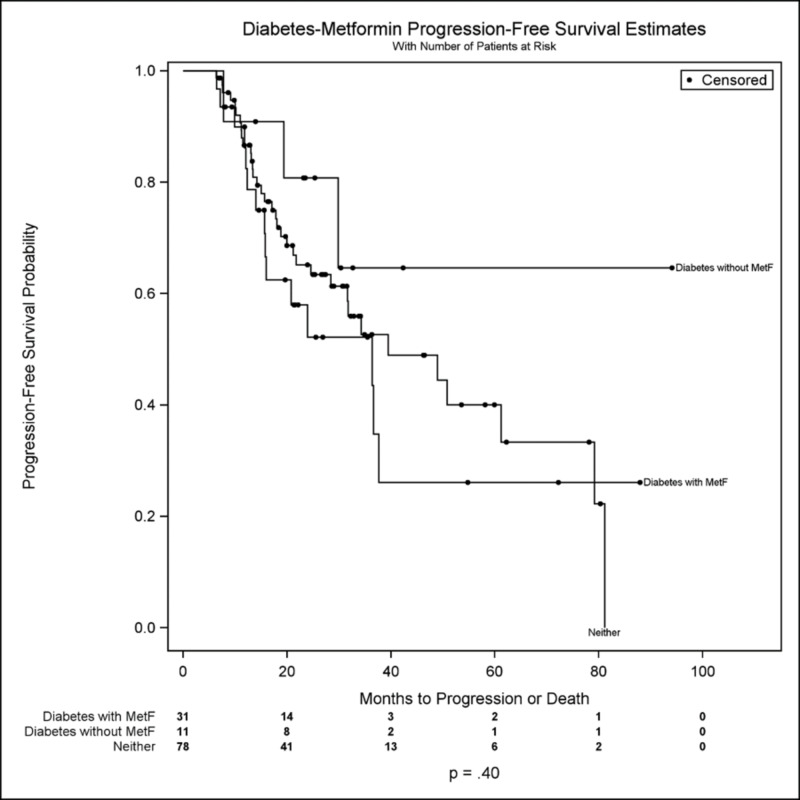
Probability of progression-free survival among diabetic patients not on metformin (Diabetes without MetF), diabetic metformin-users (Diabetes with MetF), and non-metformin users (Neither).

**Figure 2:**
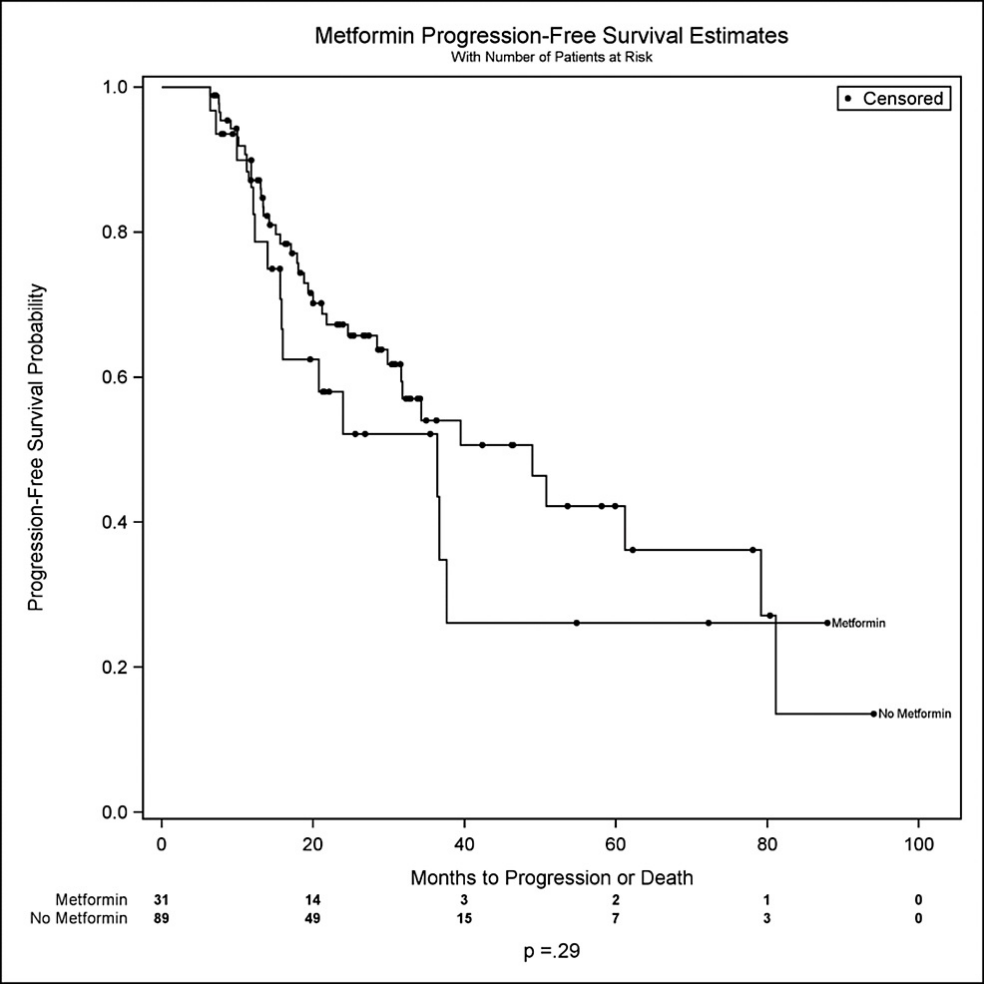
Probability of progression-free survival between metformin-users (Metformin) versus non-metformin users (No Metformin)

Median PFS for diabetic patients on metformin was 36.4 months versus 39.5 months for non-diabetic patients (figure 1); the median PFS for diabetic patients not on metformin was unobserved. In this data sample, the progression-free survival distributions for these three cohorts were comparable (overall p = 0.40). Further, there was no meaningful difference in progression-free survival between all metformin-users (Median PFS = 36.4 months) versus non-metformin users (Median PFS = 48.9 months; p = 0.29) (See figure 2). On univariable analysis of PFS, male sex (p = 0.03) and increasing tumor size (p = 0.01) were hazardous (tables 2-3), but there was no significant effect of metformin use on PFS (p = 0.34).

**Table 2:** Univariable progression-free survival estimates.

	Hazard Ratio	95% Confidence Interval		p
		Lower	Upper	
Metformin: Yes versus No	1.34	0.74	2.44	.34
Diabetes Yes versus No	1.00	0.56	1.77	.99
Diabetes-Metformin				.35
Diabetes with Metformin vs Neither	1.24	0.68	2.28	.49
Diabetes without Metformin vs Neither	0.50	0.15	1.64	.25
Diabetes with Metformin vs Diabetes without Metformin	2.48	0.72	8.58	.15
Tumor Size (unit = 1mm)	1.38	1.08	1.78	.01
T-Stage 2a or 2b vs 1a or 1b	1.48	0.80	2.73	.21
Sex Male versus Female	2.02	1.07	3.81	.03
Age (unit = 1 year)	1.01	0.98	1.04	.44
Treatment Era After 2009 versus Before 2009	0.67	0.37	1.22	.19
Karnofsky Performance Score (unit = 1 point)	0.97	0.95	1.001	.059
Pathology (versus No Pathology)				.75
Adenocarcinoma	1.06	0.51	2.21	.88
NSCLC NOS	1.43	0.61	3.37	.41
Squamous	1.38	0.65	2.95	.40
Dose: 60 Gy x 5 versus 50 Gy x 5	1.43	0.64	3.20	.39
Note: Valid N = 120 with 53 events. NSCLC NOS = Non-small cell lung cancer not otherwise specified				

**Table 3:** Progression-free survival summary statistics

	Total (N = 120)	Progression or Mortality	
		No (n = 67)	Yes (n = 53)
Metformin	31 (26%)	16 (24%)	15 (28%)
Diabetes	42 (35%)	24 (36%)	18 (34%)
Diabetes-Metformin			
Neither	78 (65%)	43 (64%)	35 (66%)
Diabetes without metformin	11 (9.2%)	8 (12%)	3 (5.7%)
Diabetes with metformin	31 (26%)	16 (24%)	15 (28%)
T-Stage			
1A or 1B	95 (79%)	56 (84%)	39 (74%)
2A or 2B	25 (21%)	11 (16%)	14 (26%)
Male Sex	80 (67%)	40 (60%)	40 (75%)
Treatment Era			
Before 2009	23 (19%)	5 (7.5%)	18 (34%)
After 2009	97 (81%)	62 (93%)	35 (66%)
Pathology			
Adenocarcinoma	40 (33%)	24 (36%)	16 (30%)
Squamous	31 (26%)	17 (25%)	14 (26%)
NSCLC NOS	17 (14%)	8 (12%)	9 (17%)
No pathology	32 (27%)	18 (27%)	14 (26%)
Deceased	49 (41%)		
Mean Tumor Size (SD)	2.24 (1.04)	2.05 (0.91)	2.48 (1.15)
Mean Age (SD)	71.38 (9.09)	71.07 (8.20)	71.77 (10.17)
Median KPS (IQR)	80 (70 – 90)	80 (70 – 90)	80 (70 – 90)
Dose			
50 Gy x 5	102 (85%)	56 (84%)	46 (87%)
60 Gy x 5	18 (15%)	11 (16%)	7 (13%)
Note: KPS = Karnofsky Performance Score. NSCLC NOS = Non-small cell lung cancer not otherwise specified. SD = Standard deviation of the mean. IQR = Interquartile range.			

Finally, in this data sample, the grade 2 toxicity rate between metformin users and non-users was comparable (p = 0.51). There were no grade ≥3 acute or chronic toxicities in either group.

## Discussion

Interest in the use of metformin as a potential adjunctive antineoplastic agent has grown over the past decade, fueled by preclinical data demonstrating multiple antitumoral effects and retrospective clinical data demonstrating the reduced risk of cancer incidence as well improved PFS and OS in several disease sites [4,6-13]. In lung cancer, metformin has similarly been shown to exert antineoplastic effects and radiosensitization [11,12].

**Figure 3:**
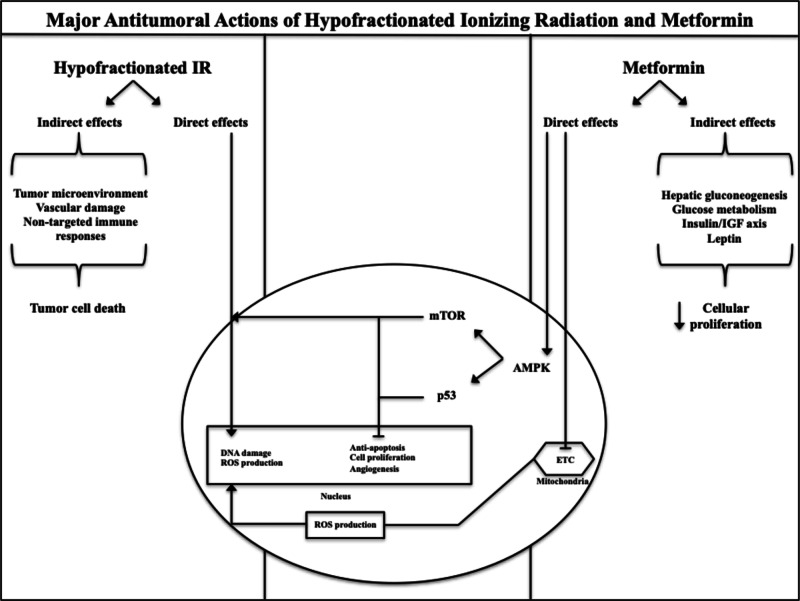
Putative cellular mechanisms of hypofractionated radiotherapy and metformin. ETC: Electron transport chain; IR: Ionizing radiation; mTOR: Mechanistic target of rapamycin; AMPK: AMP-activated protein kinase; IGF:  Insulin-like growth factor; ROS: Reactive oxygen species

There are multiple postulated mechanisms of metformin's anti-cancer effects (Figure 3). First, metformin activates adenosine monophosphate kinase (AMPK), which inhibits the mammalian target of Rapamycin (mTOR) and its downstream tumour proliferation effects [9, 21]. Second, metformin inhibits hepatic gluconeogenesis and stimulates peripheral insulin sensitivity, thus inhibiting the tumour-specific stimulatory effects of hyperinsulinemia [22]. Finally, metformin inhibits the mitochondrial electron transport chain, increasing intracellular reactive oxygen species, which decreases the overall fraction of hypoxic tumour cells and provides a putative mechanism for radiosensitization [23]. While these mechanisms may explain the beneficial role of metformin in the setting of conventionally fractionated radiotherapy, it is less evident in hypofractionated radiotherapy. There has been debate in the literature regarding the radiobiological mechanism of hypofractionated radiotherapy [24]. Specifically, it has been hypothesized that a major etiology of cell kill in hypofractionated radiotherapy is derived from indirect cellular effects, including disruption of tumour vasculature as well as local immunologic effects [25].

Ahmed et al. retrospectively analyzed the effect of metformin use on 166 patients treated with chemoradiotherapy for locally advanced NSCLC [11]. With a median follow-up of 17 months, there was no significant difference in LC, OS or PFS between metformin users and non-users. Wink et al. similarly evaluated metformin's effect on outcomes in locally-advanced NSCLC treated with chemoradiotherapy [12]. In their cohort of 682 patients, they found significantly improved distant metastasis-free survival and PFS, but no difference in local recurrence-free survival or OS. While these studies are intriguing, and one study demonstrated a clinically measurable interaction of metformin with chemoradiotherapy in lung cancer, they were in the setting of conventionally fractionated radiotherapy. We await data from ongoing and accrued clinical trials to determine the role of metformin in this setting.

Clinical data on metformin use in the setting of hypofractionated radiotherapy in lung cancer patients is scarce. We hypothesized that the addition of metformin to lung SBRT would improve LC in the treatment of early-stage NSCLC. However, in our retrospective sample of patients treated with SBRT for early-stage NSCLC, metformin had no significant PFS effect.

Indirect cellular effects induced by SBRT may explain the differential results of metformin in the setting of conventional fractionation vs SBRT. However, much remains unknown regarding the precise circumstances in which metformin may exert clinically significant antitumoral effects in the setting of radiotherapy. Specifically, metformin's radiosensitizing effects are context-dependent. Preclinical data have shown radiosensitization and radioprotective effects of metformin within the same lung cancer cell lines [26]. Additionally, while metformin has been postulated to potentiate radiotherapy by increasing available oxygen substrate for reactive oxygen species (ROS) within cells, it has also been shown to reduce basal ROS production [27].

Metformin use may alter the tumour microenvironment such that the indirect vascular effects of hypofractionated radiotherapy are less effective, and in fact may be attenuated. Preclinical data have demonstrated significant heterogeneity in NSCLC tumour metabolism [28]. It seems likely that the sub-classification of NSCLC cell types, which has progressed from histological groups to molecular subtyping, will continue to include the therapeutically relevant sub-classification scheme of metabolic sub-typing. However, more data is needed to elucidate the complex interactions between NSCLC tumour metabolism, hypofractionated radiotherapy, and metformin.

In the setting of hypofractionated radiotherapy, to our knowledge, there has been only one study to investigate the effect of concurrent metformin use with hypofractionated radiotherapy. Harder et al. reported in a retrospective series of early-stage lung cancer patients treated with SBRT that metformin users had significantly lower rates of regional control, distal control, and disease-free survival, and a trend toward poorer LC and OS [29]. Interestingly, their study did not demonstrate an association between any type of failure and other anti-hyperglycemic medications, including sulfonylureas, sitagliptin, insulin, or thiazolidinone. Taken together, our retrospective series suggest that the interface between ionizing radiation, metformin, tumour metabolism, tumour hypoxia, and tumour microenvironment may be fundamentally different in the setting of conventional vs hypofractionated radiotherapy.

This study is limited by its retrospective design and small sample size, which comprised all available patients treated with SBRT for early-stage NSCLC at the time of the analysis. There are broad and complex interactions between diabetes, obesity, metabolism, and metformin in the context of lung cancer, which are poorly understood in the setting of hypofractionated radiotherapy. Further, diabetes and obesity may be significant confounding factors in terms of disease control. Future studies are needed to understand further the degree of diabetes control, BMI, and other potentially confounding metabolic and co-morbid conditions.

In the setting of early-stage NSCLC, an M.D. Anderson Cancer Center trial investigates whether the addition of metformin concurrent to SBRT is more effective than radiation therapy alone (NCT02285855) [30]. Importantly, this trial plans to not only assess tumour response following treatment, but it will compare treatment outcomes between subgroups defined by tumour mutational status.

## Conclusions

Previous data have suggested conflicting interactions between metformin use concurrent with conventionally fractionated radiotherapy, with some retrospective data suggesting a synergistic benefit. However, our study suggests an alternate interaction with metformin and an ultra hypofractionated radiotherapy regimen as with SBRT. For this reason, we look with anticipation toward the results of prospective trials that seek to evaluate this question with both larger sample size and stratify the effect of concurrent metformin with SBRT for NSCLC based on tumour mutational status.
